# Anxiety Levels Among Women Undergoing Mammogram Screening

**DOI:** 10.3390/curroncol32030160

**Published:** 2025-03-12

**Authors:** Wedad M. Almutairi, Salwa Hassan Alzahrani

**Affiliations:** 1 Maternity and Child Department, Faculty of Nursing, King Abdulaziz University, Jeddah 21551, Saudi Arabia; salzahrani1486@stu.kau.edu.sa; 2 Executive Administration of Public Health, Al-Baha Health Cluster, Al-Baha 65784, Saudi Arabia

**Keywords:** breast cancer, mammogram, psychological barriers, anxiety, contributing factors

## Abstract

Breast cancer is the leading cause of death among women around the world. In Saudi Arabia, breast cancer remains a challenging health problem which accounted for 31.7% of all cancer cases in Saudi females, with an age-standardized incidence rate (ASR) of 29.7 and an estimated death rate of 9.67 per 100,000 Saudi women in 2022. Early detection is confirmed to be the best practice for better prognosis. Mammography screening is one of the most effective methods of early detection. However, anxiety about mammogram screening may affect early detection. There is a lack of studies regarding the psychological impact, such as anxiety, on women who undergo mammogram screening in Saudi Arabia. **Thus, the aim of this study** is to assess the level of anxiety and its contributing factors in women who undergo mammogram screening at Breast Cancer Screening Centers in Saudi Arabia. **Design:** A descriptive cross-sectional design was conducted. **Setting:** Mammogram clinics in East Jeddah Hospital and King Fahad Hospital. **Sample:** A convenience sample of 218 was collected. The data were collected from March 2023 to July 2023. **Tools:** The Penn State Worry Questionnaire (PSWQ) and the Psychological Consequence Questionnaire (PCQ). **Result:** Based on the PSWQ scale, the total mean of the anxiety level in our sample was mild anxiety (mean = 43.4, SD = 11.4). Based on the PCQ, the results demonstrated that physical, emotional, and social factors were significantly associated with the anxiety level, respectively (r = 0.4, *p* = 0.001; r = 0.489, *p* = 0.001; r = 0.337, *p* = 0.001). **Conclusions and recommendations:** Saudi women showed mild anxiety levels during mammogram screening. The physical, emotional, and social factors impact the anxiety level in women undergoing mammogram screening, which might explain the low rate of mammogram screening adherence in Saudi Arabia.

## 1. Introduction

One of the main causes of cancer-related deaths is breast cancer, which is also the most often diagnosed cancer among women globally. Breast cancer affected over 2.3 million women in 2020, accounting for 11.7% of all cancer cases worldwide. Approximately 685,000 deaths worldwide were attributed to breast cancer in 2020, which accounted for 6.9% of all cancer-related deaths [1,2]. In Saudi Arabia, breast cancer remains a challenging health problem which accounted for 31.7% of all cancer cases in Saudi females, with an age-standardized incidence rate (ASR) of 29.7 and an estimated death rate of 9.67 per 100,000 Saudi women in 2022 [3,4]. The early identification of breast cancer markedly enhances prognosis and survival rates. Multiple studies have shown that cancers detected at an early stage are more responsive to treatment, resulting in improved outcomes. Research demonstrates that the average mortality risk from breast cancer after five years of diagnosis has diminished from 14% to 5% since the 1990s, primarily attributable to early detection and treatment advances. There are many methods used for early detection of breast cancer; these include clinical breast examinations, mammogram screening, genetic screening, molecular diagnostics, ultrasonography, and magnetic resonance imaging; nevertheless, the three essential screening tests are clinical breast examinations, mammogram screening, and ultrasound. However, mammography is recognized to be the gold standard for the early detection of breast cancer, especially for asymptomatic women. A study by Welch et al. published in the New England Journal of Medicine investigates the impact of mammography screening on breast cancer outcomes, emphasizing tumor size distribution, overdiagnosis, and mortality reduction. Screening markedly enhanced the identification of minor malignancies (<2 cm) from 36% to 68%, concurrently decreasing the incidence of large tumors (≥2 cm) from 64% to 32%. However, most additional small cancers identified were overdiagnoses, with 132 out of 162 cases per 100,000 women being tumors unlikely to advance to clinical relevance [5]. Eby, Ghate, and Hooley review the recent observational studies findings on the effects of mammography screening and early diagnosis on breast cancer outcomes. They found that screening reduces breast cancer mortality, advanced tumors, and invasive therapies. Also, screening programs reduced breast cancer mortality by 48–51% in women aged 50–79 who had regular screening. Mammograms can discover breast cancer early, which reduces the need for mastectomies and chemotherapy and radiation therapies [6].

Moreover, Autier et al. (2024) conducted a systematic review including 18 cohort studies to evaluate the impact of mammography screening on breast cancer-specific and overall death rates. The results demonstrated that women who underwent mammography screenings experienced a 45% reduction in breast cancer mortality (Relative Risk [RR]: 0.55, 95% Confidence Interval [CI]: 0.50–0.60) and a similar 46% drop in overall mortality (RR: 0.54, 95% CI: 0.50–0.58) [7]. Both the American College of Obstetricians and Gynecologists (ACOG) and the World Health Organization (WHO) recommend mammographic screening. The ACOG recommends that average-risk women should start mammogram screening at age 40; supporting that early detection can significantly reduce breast cancer mortality rates with adjustment of screening frequency through shared decision-making, it highlights the necessity of considering patient-specific factors. The WHO similarly encourages mammogram screening as a critical element of early detection programs, especially in settings with adequate healthcare services [8,9].

However, anxiety linked to mammography screenings is a significant issue that might affect women’s interest in breast cancer detection and adherence. Recent research has examined the psychological impacts of mammography and its influence on screening practices. Psychological factors such as fear and anxiety are confirmed to be one of the barriers to undertaking mammogram screening [10]. Moreover, anxiety before the screening leads to negative impacts on the experiences of women undergoing a mammogram [11]. Jaafar Sidek et al. conducted a study aimed at assessing the prevalence of anxiety in women undergoing mammography and identifying the variables leading to anxiety. The study design was a cross-sectional analysis including 213 women who filled out self-administered questionnaires. The average age of the participants was 57 years, most of them of Malay origin, possessing secondary education, and classified as low-income. The findings indicate that 41.8% of the women experienced moderate to high anxiety levels, mainly due to worry about the mammography results (69%). Additional notable anxiety-inducing causes were apprehensions regarding procedural discomfort (58%) and anticipated adverse radiation effects (56.3%). In contrast, knowledge of the technique markedly lowered anxiety levels. A statistical study revealed no significant correlations between anxiety and socio-demographic or clinical variables, including age, marital status, educational attainment, or income. A significant positive association was identified between state and trait anxiety levels (r = 0.568, *p* < 0.001), indicating that those with high baseline anxiety are predisposed to increased situational worry.

The study indicated that minimizing patient worries, especially anxiety about outcomes, by enhanced communication and education on the treatment reduced anxiety [12].

In Saudi Arabia, historically, breast cancer screening rates have been low. A 2015 survey indicated that just 8% of women had participated in mammography screening. Recent research from 2023 reveals that 69% of Saudi women aged 40 and older had never undergone a mammogram, while the screening is free for all citizen aged 40 and above in all public hospitals with orders from any primary healthcare providers. These statistics highlight the necessity for increased knowledge and accessibility to increase participation in breast cancer screening initiatives, especially the ministry of health initiation programs, to reduce the mortality rate of breast cancer [13,14]. Furthermore, increasing knowledge of mammogram screening and providing accurate information on its advantages and low risks were suggested as measures to promote participation and adherence in breast cancer screening programs.

However, there is a lack of studies regarding the psychological impact, such as anxiety, on women who undergo mammogram screening in Saudi Arabia. Therefore, our study aim is to assess the level of anxiety and the contributing factors in women who undergo mammogram screening at Breast Cancer Screening Centers in Saudi Arabia. Our study aim was met by answering the following research questions: 1—What is the level of anxiety before a mammogram screening appointment? 2—What are the contributing factors affecting anxiety levels in women undergoing mammogram screening?

## 2. Materials and Methods

This study applied a descriptive cross-sectional design. The data were collected from March 2023 to July 2023 in two mammogram clinics: East Jeddah Hospital and King Fahad Hospital in Jeddah, Saudi Arabia. East Jeddah Hospital has a capacity of 450 beds and handles an average of 200 mammography cases monthly, while King Fahad Hospital has 650 beds and maintains an average of 230 mammogram cases monthly. The mammogram clinics in both sites are equipped with modern diagnostic equipment and staffed by the nurses, radiologists, and physicians who run the clinics. These settings were selected for accessibility for the researchers.

Sampling and sample: The researchers used a convenience sampling method, including women aged 35 to 65 years who were visiting mammograms clinics for early breast cancer screening. Women receiving mammograms for diagnostic reasons or follow-up after a breast cancer diagnosis were excluded. The sample size was calculated using the Power analysis method with a 5% margin of error and a 95% confidence level, resulting in sample size of 218.

Study tools: The data were collected using two validated tools. Penn State Worry Questionnaire (PSWQ): This 15-item scale measures level of anxiety with values ranging from 16 to 80. The scores were classified into severity ranges: ≤29 (no anxiety), 30–52 (mild anxiety), 53–65 (moderate anxiety), and ≥66 (severe anxiety) [15]. Psychological Consequences Questionnaire (PCQ): This instrument evaluated the physical, emotional, and social aspects of anxiety associated with mammogram visits. It included questions about trouble sleeping, emotional distress, and changes in social interactions [16]. Both instruments were translated into Arabic and approved by an accredited agency; then, the translated versions were pilot tested with a small subset of the target population to ensure their reliability and cultural relevance before full implementation.

Ethical approval was obtained from the committee of postgraduate studies at the nursing faculty at King Abdulaziz University and from the studies committee in the directorate of health affairs in Jeddah city.

The data were structured with Microsoft Excel and analyzed with IBM SPSS software (version 26). Descriptive statistics, including mean, standard deviation, frequency, and percentages, were used to describe the study variables. Independent *t*-tests and ANOVA were used to assess significant differences in anxiety levels among the study variables. Correlation tests were performed to examine the correlations between anxiety ratings and their contributing factors. The statistical significance was established at a *p*-value of ≤0.05. To maintain confidentiality, all the electronic data were stored on a secure computer with password protection, while hard copies were locked in a cabinet in the principal investigator’s office. Data cleansing and double entry validation ensured accuracy. Any missing or inaccurate entries were identified and corrected during the data cleaning process.

## 3. Results

This study sample included 218 women who had mammogram screening: 44.4% from East Jeddah Hospital and 55.6% from King Fahad Hospital.

### 3.1. Sample Characteristics

Table 1 shows the demographic and clinical characteristics of the participants included in this study. A total of 218 women fully answered the research questionnaire. The distribution of women by reported age were 44.0% aged 35–40 years, 37.2% aged 41–50 years, and a total of 42 women greater than 50 years old. The vast majority of participants were Saudis (95.4%), and only 4.6% of women were non-Saudis. The marital status distribution of women revealed that most of them were married (63.3%); the percentage of unmarried was 22.5%, and the remaining (14.2%) were divorced and widowed. The results of educational level distribution reported a high percentage of women with university level (61.5%), secondary education (27.5%), and about 10% of women had an intermediate or lower level of education. About 75% of women reported having children. The results showed that more than half of the women included in this study were unemployed, and most of the participants (79.4%) had no family history of cancer. There was no association between the demographics of the sample and anxiety levels, Table 2.

### 3.2. RQ1: What Is the Level of Anxiety Before a Mammogram Screening Appointment?

**Figure 1:**
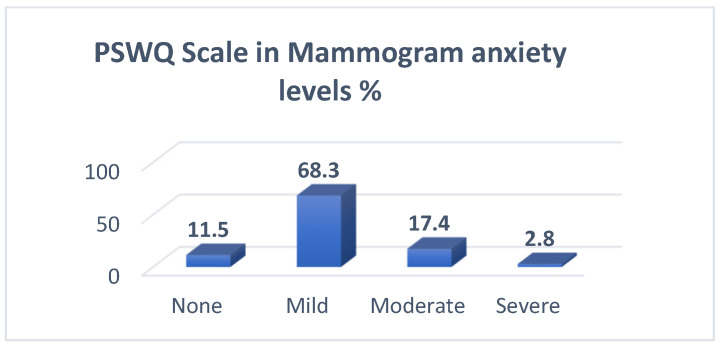
PSWQ scale of Mammogram Anxiety Levels.

To answer this question, the PSWQ scale was used, the results are shown in Table 3, and the mean and SD of the scale items were calculated. Based on the scale categorization, the total mean of anxiety level in our sample demonstrated mild anxiety (mean = 43.4, SD = 11.4). Two scale items had the highest mean of 3.19, which are “I do not tend to worry about things” and “I never worry about anything”, followed by the item of “I find it easy to dismiss worrisome thoughts”. The lowest item mean was 2.62, found in “Once I start worrying, I cannot stop”, followed by the item of “As soon as I finish one task, I start to worry about everything else I have to do”. The overall mean of the scale was 43.4± 11.4. The distribution of the PSWQ scale by categories of anxiety shows that most women (68.3%) had mild mammogram anxiety, 17.4% of women had moderate anxiety, and 2.8% had severe mammogram anxiety (Table 4, Figure 1).

### 3.3. RQ2: What Are the Contributing Factors Affecting Anxiety Levels in Women Undergoing Mammogram Screening?

To answer this question, the Pearson correlation analysis test was used on the PCQ scale, and the results demonstrated that physical factors were significantly associated with anxiety level (r = 0.4, *p* = 0.001), emotional factors were significantly associated with anxiety level (r = 0.489, *p* = 0.001), and social factors contributed significantly to the women’s anxiety level before screening (r = 0.337, *p* = 0.001).

**Figure 2:**
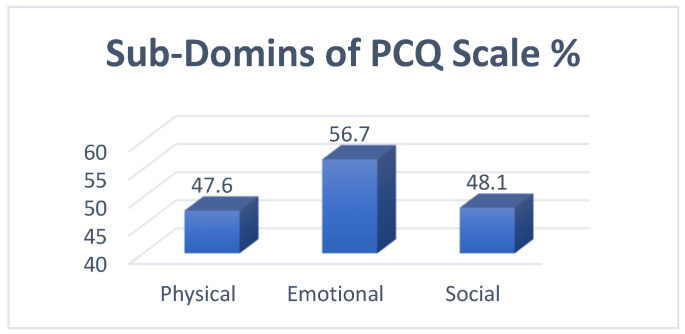
Sub-domains of mammogram anxiety scale.

The application of the PCQ scale in mammogram anxiety reported the highest item mean of 1.87 for “Keeping things from those close to you”, followed by “Felt under strain” and “Felt worried about future”, with means of 1.84 and 1.83, respectively. The lowest mean was 1.33 for “Difficulties meeting work”, followed by “Difficulties doing normal things” and “Noticeable withdrawing from those who are close”, with means 1.34 and 1.35, respectively. The overall scale mean was 18.6 ± 8.47. (Table 5). The sub-domains of the PCQ scale revealed that the emotional domain had the highest mean percentage of mammogram anxiety (56.7%), followed by the social domain (48.1%) and the physical domain (47.6%) (Table 6, Figure 2).

Table 7 above presents the results of the correlation analysis between the PSWQ scale and each domain of the PCQ scale. The correlation coefficients between the PSWQ scale and the physical, emotional, and social domains were 0.400, 0.489, and 0.337, respectively. The correlation results were significant at 1%, indicating a positive and significant relation between the PSWQ responses and each item of the PCQ scale. The highest correlation for the PSWQ was found with the emotional domain of the PCQ scale. Moreover, strong positive and significant correlations were identified between the sub-domains of the PCQ scale.

## 4. Discussion

The anxiety level before mammogram screening among most women in our study was mild, 68.3%, which is close to a similar study conducted in Turkish by ÇELİK et al. [17]; they found that the level of anxiety before a mammogram score indicated low to moderate distress. That is relevant to a study conducted by Brédart et al., in Paris, France, [18] and, also, to a study conducted to determine anxiety and depressive symptoms and missing breast cancer and cervical screening by Zhang et al. [19]. A low level of anxiety was seen in Spain, according to Aguirre-Camacho et al. [20]. However, risk communication significantly decreased the anxiety levels of women with high baseline levels of anxiety, as found in research conducted by Xie et al. in Athena [21]. Also, in our study, the physical consequences represent 47.6% of all the domains of the PCQ, which is close to a similar study conducted in Sydney by Sherman et al.; they found that women considered the experience to be moderate in terms of its positive consequences [22]. In Chicago, a study conducted by Tejeda et al. showed that the physical consequences formed 31.47%. Tejeda et al.’s study found that emotional consequences formed 33.64% and found that the social consequences formed 23.5% [22,23].

Implications: In practice, the findings of this study can be used by healthcare providers to improve their understanding of the contributing factors that can increase women’s anxiety about mammogram screening. Also, it can help nurses and radiologists in identifying women with specific factors who might be at risk for increased anxiety that can affects women’s decisions to undertake mammogram screening. This study also provides an opportunity for healthcare providers to incorporate nursing education into their plans for reducing anxiety disorders. Last, they can develop a new program focused on women who have scheduled mammogram screenings to raise awareness of anxiety disorders. Also, hospitals must have policies that are geared toward minimizing anxiety levels.

Our study has several limitations, including that the data of our study were collected from two sites only, which affected the generalization of our findings. This study’s findings were able to provide an independent scientific explanation regarding the researcher’s inquiries. This study’s participants were all women from KFH and EJH. Since most of them were from Jeddah, the findings might not represent the entire population of Saudi Arabia. Another limitation of our study is the use of general anxiety measurement tools, which may not capture distinctions specific to mammography-related anxiety as effectively as the specialized instruments used in some comparative studies. This difference could influence the direct comparability of our findings with those studies that employed mammography-specific scales. Future research should consider integrating both types of instruments to enhance the accuracy and relevance of findings.

Research Recommendations: Further research on anxiety levels before mammogram screening and the contributing factors affecting anxiety in Saudi Arabia should be undertaken with a larger sample size including different regions with heterogeneity in the sample. Also, future studies could explore the levels of anxiety in women who abstain from breast cancer screenings and develop targeted interventions to encourage their participation. This would provide a more comprehensive understanding of how anxiety influences screening behavior across different populations.

Educational Program Recommendations: Various workshops for nurses and healthcare providers in mammogram clinics are needed to address the issues related to anxiety disorders. Educational materials for women, sent to women with mammogram screening appointments, are essential. Nurses and radiologists should be motivated to develop further research on the psychological barriers to mammogram screening to support evidence-based research.

## 5. Conclusions

The physical, emotional, and social factors play a critical role in increasing or minimizing the level of anxiety in women undergoing mammogram screening, and the majority of Saudi women who undergo mammogram screening have a mild anxiety level, which might contribute to the low rate of screening adherence in Saudi Arabia.

## Figures and Tables

**Table 1:** Demographic characteristics of participants.

Variable	N = 218	%
Age		
35–40	96	44.0
41–50	81	37.2
51–60	38	17.4
60+	3	1.4
Marital status		
Married	138	63.3
Unmarried	49	22.5
Divorced	19	8.7
Widowed	12	5.5
Educational level		
Uneducated	1	0.05
Primary	10	4.6
Intermediate	13	6.0
Secondary	60	27.5
University	134	61.5
Having children		
Yes	162	74.3
No	56	25.7
Employment status		
Employed	100	45.9
Unemployed	118	54.1
Breast cancer family history		
Yes	45	20.6
No	173	79.4

**Table 2:** Demographic and clinical characteristics of participants by PSWQ scale.

Variable	Mean	SD	*p*-Value
Age			
35–40	44.2	10.3	0.642
41–50	42.6	12.7	
51+	43.2	11	
Nationality			
Saudi	43.3	11.3	0.52
Non-Saudi	45.7	13	
Marital status			
Married	43.5	11.3	0.963
Unmarried	42.7	11.4	
Divorced	44.2	13.9	
Widowed	43.9	8.6	
Educational level			
Primary and uneducated	45.4	12.4	0.41
Intermediate	42.9	8.7	
Secondary	41.4	10.7	
University	44.2	11.7	
Having children			
Yes	43.6	11.4	0.75
No	43	11.3	
Employment status			
Employed	43.8	12.1	0.638
Unemployed	43.1	10.7	
Breast cancer family history			
Yes	43.5	12.1	0.96
No	43.4	11.2	

**Table 3:** PSWQ scale of mammogram anxiety.

Item	Mean	SD
If I do not have enough time to do everything, I do not worry about it.	2.98	1.33
My worries overwhelm me.	2.87	1.41
I do not tend to worry about things.	3.19	1.33
Many situations make me worry.	2.84	1.41
I know I should not worry about things, but I just cannot help it.	2.92	1.43
When I am under pressure, I worry a lot.	2.84	1.45
I am always worrying about something.	2.87	1.37
I find it easy to dismiss worrisome thoughts	3.14	1.31
As soon as I finish one task, I start to worry about everything else I have to do.	2.68	1.33
I never worry about anything.	3.19	1.34
When there is nothing more I can do about a concern, I do not worry about it	3.05	1.27
I have been a worrier all my life	2.76	1.26
I notice that I have been worrying about things	2.71	1.29
Once I start worrying, I cannot stop	2.62	1.32
I worry all the time.	2.74	1.41
**Total Score**	**43.4**	**11.4**

**Table 4:** PSWQ scale of mammogram anxiety levels.

Level	N	%
None (<29)	25	11.5
Mild (30–52)	149	68.3
Moderate (53–65)	38	17.4
Severe (66 and above)	6	2.8

**Table 5:** PCQ scale of mammogram anxiety.

Item	Mean	SD
Had trouble sleeping	1.63	0.94
Experienced a change in appetite	1.42	1.00
Been unhappy	1.57	1.00
Been scared	1.55	0.97
Felt nervous	1.72	1.01
Felt under strain	1.84	0.89
Keeping things from those close to you	1.87	1.05
Taking things out on other	1.10	0.97
Noticeable withdrawing from those who are close	1.35	1.02
Difficulties doing normal things	1.34	1.05
Difficulties meeting work	1.33	0.98
Felt worried about your future	1.83	1.04
**Total Score**	**18.6**	**8.47**

**Table 6:** Sub-domains of PCQ scale of mammogram anxiety.

Domain	Items	Min	Max	Mean	SD	%
Physical	4	0	12	5.72	2.38	47.6
Emotional	5	0	15	8.51	3.95	56.7
Social	3	0	9	4.33	3.07	48.1

**Table 7:** Correlation between PSWQ scale and domains of PCQ scale.

		PSWQ	Physical	Emotional	Social
PSWQ	Pearson Correlation	1	0.400 **	0.489 **	0.338 **
	Sig. (2-tailed)		0.000	0.000	0.000
	N	218	218	218	218
Physical	Pearson Correlation	0.400 **	1	0.761 **	0.688 **
	Sig. (2-tailed)	0.000		0.000	0.000
	N	218	218	218	218
Emotional	Pearson Correlation	0.489 **	0.761 **	1	0.656 **
	Sig. (2-tailed)	0.000	0.000		0.000
	N	218	218	218	218
Social	Pearson Correlation	0.338 **	0.688 **	0.656 **	1
	Sig. (2-tailed)	0.000	0.000	0.000	
	N	218	218	218	218

**. Correlation is significant at the 0.01 level (2-tailed).

## Data Availability

All the related data presented in the manuscript.
